# Editorial: Update on the Immune Mechanisms Against Respiratory Pathogens

**DOI:** 10.3389/fimmu.2019.01730

**Published:** 2019-07-23

**Authors:** Junkal Garmendia, Jesús Gonzalo-Asensio

**Affiliations:** ^1^Consejo Superior de Investigaciones Científicas (IdAB-CSIC)-Gobierno Navarra, Instituto de Agrobiotecnología, Mutilva, Spain; ^2^Centro de Investigación Biomédica en Red de Enfermedades Respiratorias, Madrid, Spain; ^3^Grupo de Genética de Micobacterias, Departamento de Microbiología, Medicina Preventiva y Salud Pública, Facultad de Medicina, Universidad de Zaragoza, Zaragoza, Spain; ^4^Instituto de Biocomputación y Física de Sistemas Complejos (BIFI), Zaragoza, Spain

**Keywords:** bacterial respiratory pathogens, viral airway pathogens, respiratory fungi, vaccines, antimicrobials, host immunity

Respiratory infections pose a continuous threat to humans due to their easy dissemination via aerial transmission. As a consequence, they are leading causes of mortality and morbidity worldwide. Lower respiratory tract infections (LRTI) remained the deadliest communicable diseases causing 3 million deaths worldwide in 2016 (1). Similarly, although the number of tuberculosis (TB) deaths tends to decrease, it is still among the top 10 causes of global mortality with a yearly death burden of about 1.6 million (2). The growing emergence of bacterial antibiotic resistance is a major global challenge for the coming years, and several major respiratory pathogens are included in the WHO priority list of bacteria for which new antibiotics are urgently needed (3). In terms of target population, children under the age of five are the most susceptible hosts to a plethora of respiratory pathogens. The elderly, and immunocompromised respiratory patients suffering from cystic fibrosis (CF), chronic obstructive pulmonary disease (COPD), bronchiectasis, neutrophilic asthma, or silicosis are also highly targeted by respiratory pathogens, which often accelerates the fatal progression of the underlying chronic disease. Accordingly, understanding microbial pathogenicity and host immunity against respiratory infections is essential for the rational development of new and more effective therapeutics.

This Research Topic provides an updated overview of both microbial factors and host immune mechanisms determining either the control of lung infection or the development of lung disease by key respiratory pathogens including *Mycobacterium tuberculosis* (Mtb), *Streptococcus pneumoniae, Haemophilus influenzae, Bordetella pertussis, Pseudomonas aeruginosa*, influenza virus, human metapneumovirus (hMPV), *Aspergillus* spp, and *Pneumocystis* spp. Pathogen virulence factors and/or targets for immune recognition, host factors involved in pathogen recognition and immune defense, specific roles of immune cell subsets, and promising prophylactic and/or therapeutic strategies against respiratory infections have been addressed.

Regarding pathogen virulence factors, Hsieh et al., elucidated the role of Group A *Streptococcus* (GAS) NAD-glycohydrolase in causing depletion of intracellular NAD+ storage and impairment of autophagosome acidification, which in turn facilitates GAS authophagocytic killing escape and replication inside human endothelial cells. Alcalde-Rico and co-authors showed a relationship between antibiotic resistance and virulence in *P. aeruginosa*, of particular importance in the case of chronic infections, given that overexpression of the MexCD-OprJ multidrug efflux pump extrudes 4-hydroxy-2-heptylquinoline, the precursor of the *P. aeruginosa* Quinolone Signal (PQS), leading to low PQS intracellular levels and reduced production of *quorum sensing* (QS) signal molecules, which then impairs the production of QS-regulated virulence factors including elastase, protease IV, pyocyanin, rhamnolipids, and bacterial swarming. Moreover, Nieto et al., identified hemagglutinin HA S110L mutation as a potent determinant of attenuation in a pandemic 2009 H1N1 influenza virus previously isolated from a fatal case patient which also presents a highly pathogenic mutation in the polymerase subunit PA D529N, thus indicating that combination of mutations contributes to the final phenotype of such isolate. Also from the viral perspective, Soto et al., revised current knowledge on hMPV mechanisms of immune evasion contributing to poor innate immune response and thereby affecting the adaptive immunity. In particular, G protein inhibition of the IFN-I type and viral replication, SH protein inhibition of the NF-κB pathway, M2.1 protein association with pathogenesis and viral replication, and M2.2 protein inhibition of cellular responses dependent on mitochondrial antiviral signaling (MAVS) were revised. Campillo-Navarro et al. studied the production of mast cells extracellular traps (MCETs) upon cell contact with Mtb, and found that heat-killed, but not alive, Mtb can induce DNA release by mast cells, by using a mechanism likely related to increased NADPH oxidase activity and concomitant hydrogen peroxide production in such mast cells. This study also demonstrates that Mtb catalase acts as a bacterial factor likely involved in production of MCETs. Lastly, Arias and co-authors revised the mode of action of the fungal secondary metabolite gliotoxin (GT), a well-known virulence factor of the *Aspergillus* genus. GT is the most abundant mycotoxin produced by *A. fumigatus*, it causes cell death by killing cells in the spleen, thymus, or lymph nodes, and also immunosuppression by, among others, blocking macrophages inflammatory immune response by direct killing, or by inhibiting NF-κB mediated signaling as well as phagocytosis.

In terms of innate immune determinants against respiratory pathogens, several aspects have been considered. Li et al., showed that hMPV infection strongly suppresses basal- and vitamin D induced cathelicidin antimicrobial peptide (CAMP) expression in human macrophages, by a mechanism independent of vitamin D or interferon, but mediated through repressing the expression of the transcription factor C/EBPα. Likewise, Eijkelkamp et al. analyzed the antimicrobial properties of arachidonic acid (AA), a long chain polyunsaturated free fatty acid increased in the blood of *S. pneumoniae* infected animals, and showed that AA exerts its antimicrobial activity via insertion into the bacterial membrane, resulting in altered membrane composition and increased fluidity which, however, did not increase pneumococcal susceptibility to antibiotic, oxidative, or metal ion stress. Also as part of the airways innate humoral arm, Casals et al., revised the roles of the collectin and galectin families, two types of endogenous lectins. Surfactant protein A (SP-A) and D (SP-D) are collectins secreted to the alveolar fluid by type II airway epithelial cells and to the airway lumen by Club and submucosal cells. Such collectins act by aggregating pathogens, which hinders their entry into epithelial cells and facilitates their removal by a variety of mechanisms such as mucociliary clearance or phagocytosis, promoting bacterial trapping by neutrophil extracellular traps (NETs), enhancing phagocytosis, or up-regulating expression of cell-surface receptors involved in microbial recognition. Conversely, galectins can function both inside and outside cells, their expression is altered in respiratory infections, bind to different respiratory pathogens (for example, Gal-3 binds mycolic acids of the mycobacterial cell wall and lipopolysaccharides from *Klebsiella pneumoniae*, and *P. aeruginosa*), modulate the immune response to infection, and present intracellular activities of importance for pathogens occupying subcellular compartments. Lastly, pathogen subversion of host factors has also been considered. Based on the notion that *S. pneumoniae* exploits neutrophil elastase (NE) leakage to subvert host innate immune responses, Domon et al., revealed that NE downregulates expression of multiple cytokines through cleavage of Toll-like receptors and myeloid differentiation factor 2, and cleaves inflammatory cytokines and chemokines. Moreover, NE inhibition increases inflammation and enhances bacterial clearance in a mouse model of pneumococcal pneumonia, thus suggesting its therapeutic potential.

As mentioned above, numerous chronic disease patients are often targeted by respiratory pathogens, likely accelerating the progression of the underlying chronic disease. This is a clinical problem of increasingly recognized interest, and major chronic respiratory diseases need to be jointly taken care off by respiratory physicians and specialists on infectious diseases. In this context, decreased levels of surfactant phospholipids reported in smokers and patients with COPD may indicate a role for surfactant lipids in host protection against bacterial infection. García-Fojeda et al. analyzed the effects of surfactant phospholipids on the interplay between *H. influenzae* and pneumocytes, showing that multilamellar vesicles, that constitute the tensoactive material of the surfactant, bind the pathogen preventing its self-aggregation and epithelial entry; differently, the use of small unilamellar vesicles, which are generated after inspiration/expiration cycles and are endocytosed by pneumocytes for their degradation and/or recycling, block bacterial cell invasion by inhibiting Akt phosphorylation and Rac1 GTPase activation. Of note, *in vivo* administration of the hydrophobic fraction of lung surfactant enhances bacterial clearance, thus suggesting its therapeutic potential. Su et al. reviewed the disease progression of COPD in the context of host immune cross-talk with non-typeable *H. influenzae* (NTHi), a bacterium with a critical role in COPD exacerbations. Features of the host-pathogen interplay leading to NTHi colonization and adaptation to the COPD patient lower airways environment have been thoroughly revised, with special emphasis on the altered expression and unresponsiveness of human TLRs to NTHi lipoproteins and lipooligosaccharide, or cigarette smoke negative effects in both the patients innate and adaptive immunity, altogether eventually facilitating NTHi long-term infection. On the other hand, Konečný et al., reviewed current knowledge about the impact of Mtb infection on silicosis, and about silica and Mtb co-exposure on the host immunity. Silicosis is a disease of the lower respiratory system with a frequent concomitant infection, and the co-occurrence of silica exposure, silicosis and TB has long been identified in populations exposed to silica-containing dust. Innate and adaptive cellular immune responses in silicosis and TB have been revised highlighting, among others, that macrophages preloaded with silica particles exhibit a higher number of Mtb phagocytic cells as well as higher rates of Mtb phagocytosis.

Our collection also explores host factors involved in the immune response against fungal and TB infections. *Pneumocystis* fungi cause fatal pneumonia in immunocompromised individuals. Moreover, although *Pneumocystis* pneumonia has gradually decreased in HIV patients due to antiretroviral therapy, it is responsible for increasing mortality in non-HIV patients. Li et al. provide insights into the function of IL-9 during *Pneumocystis* infection. By using a IL-9 deficient mouse, the authors demonstrate reduced fungal burden in lungs as well as stronger Th17 responses, compared to wild-type mice. Further experiments confirmed that IL-9 deficiency resulted in enhanced Th17 cell differentiation, and IL-17A neutralization resulted in increased fungal burden in IL-9 deficient mice. Regarding immunity against TB, Mpande et al. explored the presence of stem cell memory T CD4^+^ cells (T_SCM_) in TB infected humans. This T cell subset is absent in quantiferon (GFT)-negative individuals, but displays measurable, and maintained levels after QFT conversion, suggesting that primary Mtb infection induces T_SCM_ cells. Profiling of Mtb-induced T_SCM_ cells indicate higher levels of CCR5, CCR6, CXCR3, granzyme A, granzyme K, and granulysin than those found in naïve CD4+ and T_SCM_ cells. Notably, Mtb-primed T_SCM_ cells were also functional and produced IL-2, IFN-γ, and TNF-α upon antigen stimulation. Since a key feature of T_SCM_ cells is the long-term maintenance of their proliferative capacity without antigenic stimulation, understanding their functional role is expected to have valuable implications in TB vaccine development. Independently, Latorre et al. explored the use of immune markers correlating with TB latency or TB disease, as valuable tools for disease management. Once enrolled two cohorts of active and latent TB patients, expression of CD27, and CCR4 homing markers was studied in blood CD4^+^ T cells. A higher diagnostic accuracy for active TB was achieved for CD27 within IFN-γ^+^TNF-α^+^CD4^+^ T-cells in response to ESAT-6/CFP-10 stimulation, followed by CD27 and CCR4 markers within IFN-γ^+^CD4^+^ T-cells in response to purified protein derivative (PPD). In an independent study, Jaisinghani et al. sought to investigate metabolic signals leading to granulomatous inflammation in pulmonary TB, and found an association between inflammatory response and the presence of triglyceride (TG)-rich foamy macrophages in necrotic granulomas. The absence of these foamy macrophages in solid granulomas paved the way for downstream experiments to demonstrate that *in vitro* infection of macrophages with Mtb leads to increased TG production only under necrotic conditions. Notably, the human enzyme diacylglycerol O-acyltransferase (DGAT1) involved in TG synthesis appears to be responsible of this phenomenon since DGAT1 silencing resulted in suppressed expression of pro-inflammatory mediators. Lastly, Lacoma et al. reviewed a panoply of host genetic factors involved in respiratory tract infections, including ciliopathies leading to impaired mucociliary clearance, as those leading to cystic fibrosis, deficiency in alpha 1 antitrysin in some COPD patients, or disorders in humoral immunity leading to primary immunodeficiencies. In addition, single nucleotide polymorphisms (SNPs) in TLR-2, TNF-α, IL-12, IFN-γ, and their corresponding receptors are associated with increased risk of developing TB, while mutations in ICAM-3 have been linked with reduced risk of disease. Moreover, the link between treatment with different biological response modifiers and the increased risk of pneumonia, influenza, TB, *Pneumocystis*, and fungal infections of the respiratory tract has been revised.

This Research Topic also explored conventional and unconventional therapeutic interventions against respiratory pathogens. Despite the availability of vaccines and antibiotics against major players in respiratory infections, some pathogens are reluctant to be prevented by an efficacious vaccine, and the emergence of antibiotic resistance is an alarming threat. Moreover, some respiratory pathogens are prone to form biofilms that otherwise result in enhanced antibiotic resistance. Vázquez et al. reviewed respiratory pathogens fighting based on the use of phage lysins which specifically target susceptible bacteria by hydrolysing bacterial peptidoglycan, and whose major advantage is the fact that raising resistance seems to be unlikely. This strategy might circumvent current problems in infection treatment failure, further resulting more friendly with the human microbiome than currently overused antibiotics. Phage lysins are particularly active against Gram-positive pathogens (*S. pneumoniae, S. aureus*, and *S. pyogenes*) and mycobacteria, and some strategies are also being developed against Gram-negative pathogens (*P. aeruginosa, Acinetobacter baumanii*, and *K. pneumoniae*). In addition, Domenech et al. reviewed the combined use of antibodies and antibiotics to treat respiratory infections. Alteration of cell surface structures driven by antibiotics might result in increased exposure of bacterial antigens, which in turn may act as an alternative strategy to overcome multidrug resistant pathogens. Previous studies using a *S. pneumoniae* sepsis model demonstrated the synergistic action of antibodies with β-lactams and macrolides. Several antibodies are currently being tested in clinical trials against ESKAPE pathogens (*Enterococcus faecium, S. aureus, K. pneumoniae, A. baumannii, P. aeruginosa*, and *Enterobacter* spp.) and might pave the way for future combinations with existing antibiotics. Ramos-Sevillano et al. provided and update on the natural immunity against *S. pneumoniae* focused on antibody- and Th17-mediated immunity against specific pneumococcal antigens. This manuscript reviewed existing data regarding the impact of natural immunity during nasopharyngeal colonization, pneumonia or septicaemia by *S. pneumoniae*. Mechanisms involved in preventing infection seem to be dependent on the infected anatomical site, with an emphasis on antibodies during systemic infection and on Th17 CD4^+^ T cells for nasopharyngeal infection, with pneumonia seemingly a combination of these two. Increasing knowledge by collecting human data might help to understand why some patient subpopulations are at high risk of infection, or also aid to improve future vaccine strategies. Closely related, Liao et al. tried to decipher the protective mechanism of the live-attenuated pneumococcal vaccine SPY1. Intranasal administration of this vaccine to mice resulted in increased IL-12p70, IL-4, IL-5, and IL-17A, decreased the infection-associated inflammatory cytokine TNF-α, and in increased production of TGF-β1 in lung and spleen homogenates which is in turn related to up-regulation of Smad2/3 and down-regulation of the negative regulator Smad7. SPY1-specific Tregs are supposed to participate in protection by enhanced expression of the immune modulators PD-1 and CTLA-4. Gupta and Nizet exploited the notion of stabilizing the host Hypoxia-Inducible Factor-1 alpha (HIF-1α) in mesenchymal stem cells (MSCs) as a promising therapeutic approach against severe pneumonia. The use of the selective small molecule AKB-4924 resulted in longer half live of HIF-1α, reduced MSC death under cytotoxic conditions, and enhanced antibacterial capacity of MSC. Further, using a model of *E. coli* experimental pneumonia, these authors demonstrated sustained protection against mortality in mice. Regarding pertussis, Solans and Locht reviewed the key role of the respiratory mucosal immunity in protection against *B. pertussis* and exploited current knowledge to explain long-lasting immunity of BPZE1, a live-attenuated vaccine under clinical development. Upon infection, *B. pertussis* triggers production of secretory IgA (sIgA) and cellular immune mechanisms as tissue resident memory T (Trm) cells characterized by CD4^+^, CD69^+^, and/or CD103^+^ that produce IFN-γ and/or IL-17. Intranasal vaccination of mice with BPZE1 induced both sIgA and CD4+CD69+CD103+ Trm cells in the nasal mucosa, and these cells produced high levels of IL-17. These mechanisms are tough to be responsible to protect against nasal colonization with virulent *B. pertussis*. Also related to pertussis vaccination strategies, Varney et al. compared current acellular and whole-cell pertussis vaccines in terms of their ability to profile the hematopoietic stem and progenitor cell (HSPC) responses. Whole cell but not acellular pertussis vaccine resulted in expansion of HSPCs and increased circulating white blood cells. Upon infection, HSPCs from whole-cell vaccinated mice showed a faster maturation into developing B cells. Moreover, immunization with whole-cell pertussis vaccine resulted in enrichment of interferon-induced genes within HSPCs. These results might help to improve current acellular pertussis vaccines in humans. In an independent study, Thofte et al. highlight the elongation factor thermo-unstable (EF-Tu) from NTHi as a novel target for bactericidal antibodies, demonstrating that EF-Tu is surface exposed and identifying immunodominant epitopes in this protein. Anti-EF-Tu IgG detected EF-Tu on unencapsulated bacteria, considerably less EF-Tu was found at the surface of encapsulated *H. influenzae* serotype b (Hib) and *S. pneumoniae* (serotypes 3 and 4), and capsule removal facilitated EF-Tu exposure. Of note, anti-NTHi EF-Tu IgG antibodies promoted complement-dependent killing of this and other uncapsulated bacteria. Regarding treatment of patients with Ig deficiencies, Langereis et al. revised current regimes of Ig replacement therapies. Treatment of agammaglobulinemia patients includes Ig preparations that only contain IgG but lack IgA and IgM. Consequently, deficiency in these latter Ig results in lesser control of respiratory infection. Current regimes enriched in IgA and IgG including fresh frozen plasma, pentaglobin, trimodulin, IgAbulin, or purified IgA and IgM linked to a secretory component were reviewed. Lastly, Kroesen et al. used aspirin at low doses to ameliorate lung pathology in a murine model of TB, putting forward the notion that low-dose aspirin may be beneficial when combined with standard anti-TB treatment due to its anti-inflammatory effect and enhanced Th1-cell responses.

Finally, we want to express our gratitude to all the authors who have contributed to this Research Topic and to the reviewers for their timely and critical job. We hope that the reader will find this Research Topic motivating and helpful. We invite you to read the following articles and immerse yourself in the interesting world of the molecular mechanisms of host-pathogen interplay at the human airways.

## Author Contributions

All authors listed have made a substantial, direct and intellectual contribution to the work, and approved it for publication.

### Conflict of Interest Statement

The authors declare that the research was conducted in the absence of any commercial or financial relationships that could be construed as a potential conflict of interest.
